# Theoretical Analysis of the Stress Induced B-Z Transition in Superhelical DNA

**DOI:** 10.1371/journal.pcbi.1001051

**Published:** 2011-01-20

**Authors:** Dina Zhabinskaya, Craig J. Benham

**Affiliations:** UC Davis Genome Center, University of California, Davis, Davis California, United States of America; University of North Carolina at Chapel Hill, United States of America

## Abstract

We present a method to calculate the propensities of regions within a DNA molecule to transition from B-form to Z-form under negative superhelical stresses. We use statistical mechanics to analyze the competition that occurs among all susceptible Z-forming regions at thermodynamic equilibrium in a superhelically stressed DNA of specified sequence. This method, which we call SIBZ, is similar to the SIDD algorithm that was previously developed to analyze superhelical duplex destabilization. A state of the system is determined by assigning to each base pair either the B- or the Z-conformation, accounting for the dinucleotide repeat unit of Z-DNA. The free energy of a state is comprised of the nucleation energy, the sequence-dependent B-Z transition energy, and the energy associated with the residual superhelicity remaining after the change of twist due to transition. Using this information, SIBZ calculates the equilibrium B-Z transition probability of each base pair in the sequence. This can be done at any physiologically reasonable level of negative superhelicity. We use SIBZ to analyze a variety of representative genomic DNA sequences. We show that the dominant Z-DNA forming regions in a sequence can compete in highly complex ways as the superhelicity level changes. Despite having no tunable parameters, the predictions of SIBZ agree precisely with experimental results, both for the onset of transition in plasmids containing introduced Z-forming sequences and for the locations of Z-forming regions in genomic sequences. We calculate the transition profiles of 5 kb regions taken from each of 12,841 mouse genes and centered on the transcription start site (TSS). We find a substantial increase in the frequency of Z-forming regions immediately upstream from the TSS. The approach developed here has the potential to illuminate the occurrence of Z-form regions *in vivo*, and the possible roles this transition may play in biological processes.

## Author Summary

We present the SIBZ algorithm that calculates the equilibrium properties of the transition from right-handed B-form to left-handed Z-form in a DNA sequence that is subjected to imposed stresses. SIBZ calculates the probability of transition of each base pair in a user-defined sequence. By examining illustrative examples, we show that the transition behaviors of all Z-susceptible regions in a sequence are coupled together by the imposed stresses. We show that the results produced by SIBZ agree closely with experimental observations of both the onset of transitions and the locations of Z-form sites in molecules of specified sequence. By analyzing 12,841 mouse genes, we show that sites susceptible to the B-Z transition cluster upstream from gene start sites. As this is where stresses generated by transcription accumulate, these sites may actually experience this transition when the genes involved are being expressed. This suggests that these transitions may serve regulatory functions.

## Introduction

DNA often occurs in an underwound, negatively superhelical topological state *in vivo*. In bacteria, gyrase enzymes act to generate negative supercoils, while topoisomerases dissipate them. The dynamic balance between these two processes determines a basal level of superhelicity that can change according to the environmental or nutritional state of the organism [1]. In addition, RNA polymerase translocation leaves a wake of negative supercoils and generates a bow wave of positive supercoils [2]–[4]. Together these effects induce substantial amounts of superhelicity in the topological domains into which bacterial genomes are subdivided. A variety of regulatory processes in prokaryotes, including the initiation of transcription from specific genes, are known to vary with the level of superhelicity experienced by the DNA involved [5].

It has long been thought that unconstrained superhelicity was not a factor in eukaryotic genomic regulation. Eukaryotes do not commonly have negatively supercoiling gyrases while they do have relaxing topoisomerases. Also nucleosomal winding both stabilizes supercoils and could inhibit the transmission of unconstrained superhelicity. However, it is now known that substantial amounts of transcriptionally induced negative superhelicity occur upstream (i.e. 5′) of RNA polymerases in the human genome [6], [7]. A superhelix density of 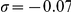 is achieved there by a single transcriptional initiation event, while divergently oriented transcription can produce superhelix densities of 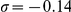 in the region between the polymerase complexes. This superhelicity extends over at least kilobase distances, hence must be transmitted either through or around nucleosomes. Kinetically, this transcription driven superhelicity is generated faster than topoisomerases act to relieve it, so it abides long enough to be able to affect subsequent regulatory processes.

The levels of negative superhelicity achieved in both prokaryotes and eukaryotes are sufficient to drive *in vivo* structural transitions to alternative DNA conformations [7], [8]. The most studied DNA transition is superhelically induced duplex destabilization (SIDD), which facilitates or creates local sites of strand separation. SIDD has been implicated in a wide variety of regulatory processes, including the initiation of transcription from specific promoters in both prokaryotes and eukaryotes [9]–[17].

Here we focus on the transition from B-form to Z-form, a left-handed double helix. When the discovery of Z-DNA was announced this transition was predicted to occur at physiologically attained levels of negative superhelicity [18]–[20]. Z-DNA has been experimentally detected at inserted Z-susceptible sites in bacterial genomic DNA both *in vitro* and *in vivo* [21]–[26].

The study of alternate DNA structures in eukaryotes is more challenging, in part because DNA superhelicity in these organisms seems not to be stable, but rather is a transient state driven by transcriptional activity. However, there is substantial indirect evidence that Z-DNA also can occur *in vivo* in eukaryotes. Z-DNA has been implicated in a variety of regulatory events relating to replication, transcription, recombination, and other biological processes [27]. For example, it has been shown that the negative torsional stress induced by polymerase translocation during transcription can stabilize Z-DNA near transcription start sites [28]. The amount of Z-DNA found in these experiments was directly related to transcriptional activity, and thus to the level of transcription-driven superhelicity. Another set of experiments studied the formation of Z-DNA in the 5′ flank of the human c-*myc* gene [29], [30]. Three Z-susceptible regions were identified near the promoters of this gene. These experimental results suggest that the regions involved transform to Z-form during c-*myc* transcription, but revert to B-form when transcription is inhibited. These experiments indicate that transcriptionally driven superhelical stresses can drive B-Z transitions in mammalian cells.

Many attempts have been made to identify proteins that bind selectively to Z-DNA. A powerful method developed by Herbert [31] led to the isolation of double-stranded RNA adenosine deaminase (ADAR1) [32], a Z-DNA binding enzyme, as well as other Z-binding proteins. It has been shown that E3L, a Z-DNA binding protein found in poxviruses, inhibits the host cell's ability to perform transcription or mount an anti-viral response when it is bound to Z-DNA near transcription start sites [33]. On this basis it was suggested that an inhibitor of E3L binding might protect against poxviral infection. Although there are some indications that Z-binding proteins may be involved in gene regulation, this remains an active area of research [27].

The Z-form helix has dinucleotide repeat units, one of which must be in the *syn*- and the other in the *anti*-conformation, with helicity of −12 base pairs per turn [34]. (The minus sign indicates the left-handedness of the helix.) The free energy required for the B-Z transition under low salt conditions has been determined for each of the ten dinucleotides [21], [35]–[39]. The Z-form is energetically most accessible for certain alternating purine-pyrimidine sequences, the most favored being 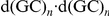, with guanine in the 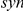 and cytosine in the 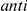 conformations. Z-formation has also been observed in 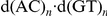 sequences, although transitions there are almost twice as costly as at GC runs. The remaining alternating purine/pyrimidine sequence, 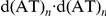, has a very high transition energy and is not normally found in Z-form. Perturbations which break the purine/pyrimidine alternation, although energetically costly, have also been observed in Z-DNA, as will be discussed below. The substantial nucleation energy for initiating a run of Z-DNA, which may be regarded as the cost of generating two junctions between B-form and Z-form, also has been determined [21], [40].

Soon after the discovery of Z-DNA several simple theoretical analyses of superhelical B-Z transitions were developed. These all assumed the simplest conditions of a single, uniformly Z-susceptible site embedded in an entirely Z-resistant background. The first such analysis simply predicted that physiological levels of negative superhelicity could drive B-Z transitions [18]. This approach was subsequently used to investigate the basic properties of these transitions, and to assess how the B-Z transition might compete with others in simple paradigm cases [19], [36], [41]–[43]. Finally, these simple theoretical approaches were applied to determine the energy parameters of the transition from experiments in which a single uniform insert (commonly 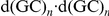) placed within a superhelical plasmid was observed to undergo transition [21], [36], [40].

In this paper we present the first method to analyze the superhelical B-Z transition in its full complexity. This method, which we call SIBZ, can calculate the B-Z transition behavior of multi-kilobase length genomic DNA sequences under superhelical stress. It specifically includes the competition for transition among all sites within the sequence. SIBZ analyzes the states available to the entire sequence, where each base can be found in either the B-conformation or as a part of a Z-form dinucleotide pair. It then uses statistical mechanics to determine the equilibrium distribution among these states. Specifically, it calculates the probability of B-Z transition for each base pair in the sequence under the given conditions. In this way it identifies the Z-susceptible regions within the sequence, and assesses how they compete at any given level of superhelicity.

SIBZ was developed by modifying the SIDD algorithm to treat the B-Z transition, as described in the following section. Several other theoretical strategies have been developed or proposed for analyzing superhelical DNA transitions, which also might have been modified for this purpose. Although a formally exact method has been suggested based on recursion relations, it was found to be too computationally inefficient to warrant development [43], [44]. So an approximate algorithm was presented in the same paper that could make base pair-specific calculations. This method has not been made available for public use or evaluation. An alternative exact algorithmic strategy also has been developed and presented [45]. Although this approach could compute transition profiles (i.e. transition probabilities for each base pair), it too was found to be too computationally cumbersome to be practical. So a more efficient approximate method based on its approach was also presented. To create SIBZ we chose to modify the SIDD approach because it has been extensively developed, optimized and implemented in this group, and it features an attractive combination of high accuracy and computational efficiency.

There have been three previous theoretical methods implemented that analyze DNA sequences to identify potential Z-DNA forming regions [35], [46]–[48]. The first method, developed by the Jovin group, seeks to identify Z-susceptible sites based solely on their sequence characteristics [46]. The energetics of transition were not considered in this approach. Another method, called *Z-Catcher*, performs a mechanical calculation, but does not consider the thermodynamic equilibrium of the system [47]. *Z-Hunt* [35], [48] uses statistical mechanics, but only calculates the propensity of each fixed region within the sequence to form a Z-helix in isolation. Since the superhelical stresses that drive B-Z transitions couple together the transition behaviors of all base pairs that experience them, these approaches do not give information about how these competitive transitions behave *in situ*.

## Methods

A DNA molecule in a topological domain is constrained by the constancy of its linking number 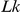, the number of times each DNA strand links through the loop formed by the other strand. The linking number of a relaxed domain is denoted 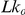. DNA domains *in vivo* are often found in a negatively superhelical state, in which 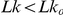. The resulting linking difference 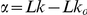, also called the superhelicity, acts to deform the molecule, in particular imposing untwisting torsional stresses. These torsional stresses can be partially or fully relieved by local secondary structural transitions to conformations that are less twisted in the right-handed sense than the B-form. This absorbs some of the linking difference as the change of twist at the transition site, which allows the balance of the domain to relax a corresponding amount. A transition becomes favored when the decrease in stress energy it provides exceeds its cost.

To perform a rigorous statistical mechanical analysis of this phenomenon one must know four things about the transition involved. First, the sequence dependence of the free energy of transition is required. Second, one needs the nucleation energy of the transition. Third, the geometry of the alternate structure determines the amount of relaxation that the transition provides. For the Z-form this is a left-handed helix with 12 base pairs per turn. Fourth, one must know the relative flexibility of the alternate structure because, if it is more flexible than the B-form, its torsional deformation may be able to relieve additional stress. This is true for strand separation because single strands of DNA are quite flexible. However, since Z-DNA is a rigid structure it is not relevant for B-Z transitions. With this information one can perform a statistical mechanical analysis of the transition in a superhelical domain having any base sequence, as described below.

An equilibrium statistical mechanical model of the superhelical strand separation transition has already been developed [45], [49], [50]. This approach, known as SIDD, has been shown to accurately predict the locations and relative amounts of destabilization of the DNA duplex experienced in defined, kilobase scale sequences at specified superhelicities [9]–[12], [15], [17], [45], [51]. In this paper, we modify the algorithmic strategy used in the SIDD method to treat stress-induced B-Z transitions (SIBZ).

### Enumeration of States

There are 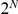 possible states available to a sequence of 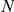 base pairs that is subject to a monomeric two-state transition (that is, one in which any individual base pair can either be in the B-form or in the alternate state). This number does not depend on whether the sequence is linear or circular. This is the situation for the strand separation transition, in which the repeat units of both states are monomeric. However, it does not hold for the B-Z transition because the repeat unit of Z-DNA is two base pairs (dimeric), while that of the B-form is a single base pair (monomeric). We will first derive an expression for the number of Z-form states available to a linear molecule of 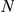 base pairs, and then use this result to determine the number of states of a circular molecule having the same length.

Let 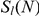 denote the number of states available to a linear molecule comprised of 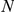 base pairs experiencing the B-Z transition. In any given state each base pair in the sequence is either a monomer (i.e. in B-form) or part of a dimeric pair with one of its neighbors (i.e. in Z-form). There are two possible arrangements for the first base pair in the sequence. It can be a monomer (i.e. in B-form), in which case there are 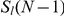 ways that the rest of the sequence can be arranged. Otherwise, it can be in a dimeric unit with the second base pair (i.e. in Z-form). In this case the disposition of the first two base pairs is determined, so there are 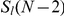 ways to arrange the rest of the sequence. Because these two alternatives are mutually exclusive and exhaust the possibilities, it follows that
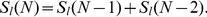
(1)Using the fact that 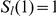 and 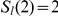, one can calculate 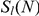 for any length 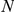 from Eq. (1). This recursion relation together with these initial conditions show that 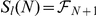, the 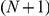
st Fibonacci number. An excellent approximation to this number for all 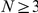 is given by
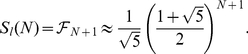
(2)This shows that the number of possible state in the linear B-Z transition grows exponentially with sequence length 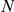, but with base equal to the golden ratio 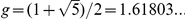, rather than base 2 as holds for transitions in which both states have monomer units.

All the states found above for a linear molecule also are available to a circular molecule of the same length. However, in this case it is also possible that base pairs 1 and 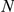 can form a dimeric unit, provided neither is already dimerized with its other neighbor. The number of states of the linear molecule in which neither base pair 1 nor base pair 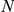 are dimerized is 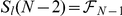. So this is the number of states of the circular molecule in which base pair 1 dimerizes with base pair 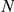. It follows that the number 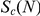 of states available to the circular molecule is
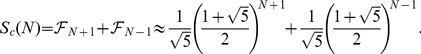
(3)


### The Equilibrium Statistical Mechanics of a Conformational Transition

In principle all states available to the molecule compete for occupancy. Once the free energy 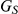 associated to each state 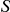 has been evaluated, the partition function 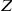 may be calculated as
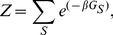
(4)where the sum is over all states, and 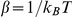, where 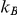 is the Boltzmann constant and 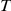 is the temperature.

At thermodynamic equilibrium the available states are weighted according to the Boltzmann distribution. That is, in the equilibrium distribution each state 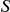 occurs with relative frequency
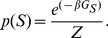
(5)This means that the occupancy of states decreases exponentially as their free energies increase, so only the relatively low energy states are significantly occupied. At equilibrium the ensemble average value of a parameter 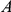, that has value 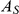 in state 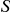, is given by
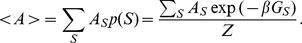
(6)The equilibrium probability 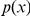 of transition for base pair 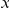 is found by averaging the parameter 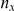 according to the above equation, where 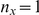 in any state where base pair 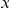 is transformed, and 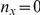 in all other states. The transition profile is the graph of 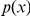 vs 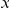. It shows the probability of transition for each base pair in the sequence under the assumed conditions. As will be shown below, this profile can change significantly as the imposed superhelix density changes.

### States and Their Energies

We consider a DNA molecule containing 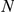 base pairs of defined sequence, on which a superhelical density 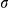 is imposed. Here 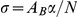, where 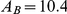 bp/turn is the helical twist rate for the B-form. A state of this molecule assigns to each base pair one of two conformations, either B-form or Z- form. This is done in a manner consistent with the dinucleotide repeat unit of Z-DNA, as described below. The residual superhelicity in that state is the linking difference remaining to stress the molecule after the change of twist consequent on transition. This includes the untwisting of the transformed base pairs from the right-handed B-form to the left-handed Z-form, together with a small amount 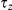 of untwisting of the two stands at each B-Z junction [21]. Thus, in a state where 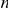 bases pairs are in the Z-form the residual superhelical linking difference is given by
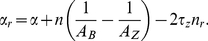
(7)Here 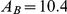 bp/turn is the twist rate for the B-helix, 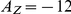 bp/turn is the twist rate of the Z-helix, and 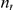 is the number of runs of Z-form DNA. (A run of transformed base pairs is defined as a maximal segment in which all base pairs are in the non-B structure.) The twist at a B-to-Z junction has been measured to be 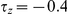 turns [21]. In our current applications the superhelicity 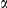 is regarded as remaining constant.

Next we determine the free energy of each state of this molecule. This is comprised of the energy cost of the transition, plus the energy of accommodating the residual superhelicity. The quadratic free energy associated to residual superhelicity [52] is given by
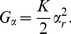
(8)Here 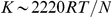 [53], [54], 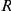 is the gas constant, and 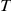 is the absolute temperature. The energy cost of the transition includes two factors. First, a nucleation energy for each run of Z-DNA is required to form the junctions between the B-form and the Z-form. This has been measured to be approximately 5.0 kcal/mol/junction, so the nucleation energy for each Z-DNA run is 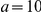 kcal/mol [21], [36], [40]. Second, the energy to transform the specified base pairs into the Z-DNA conformation is also needed. The published values of the B-Z transition free energies for all 10 dinucleotide pairs are given in the second and third columns of Table 1 [35]. The bases in each dinucleotide pair must alternate, one in *anti* and the other in *syn* conformation. As the values in the table show, the transition energy of a particular dinucleotide depends strongly on whether it is AS (5′ *anti* 3′ *syn*) or SA (5′ *syn* 3′ *anti*). A Z-Z junction occurs when adjacent dinucleotides have different *anti-syn* alternations, either (AS)(SA) or (SA)(AS). This violation is energetically costly, as shown in the last column of the Table.

**Table 1:** Sequence dependent free energies for B-Z transition.

Dinucleotide	Conformation		
	AS	SA	ZZ
CG	0.7	4.0	4.0
GC	4.0	0.7	4.0
CA = TG	1.3	4.6	4.5
AC = GT	4.6	1.3	4.5
CC = GG	2.4	2.4	4.0
CT = AG	3.4	3.4	6.3
TC = GA	3.4	3.4	6.3
AA = TT	3.9	3.9	7.4
TA	2.5	5.9	5.6
AT	5.9	2.5	5.6

Free energies are given in kcal/mole. AS and SA represent 5′-*anti* 3′-*syn* and 5′-*syn* 3′-*anti* conformations, respectively. The ZZ column shows Z-Z junction energies, as described in the text [35].

Some of the energy values shown in Table 1 are calculated estimates, while others were measured experimentally [21], [35], [37]–[39]. The energies shown can be evenly divided between the two base pairs involved, whether in a dinucleotide or in a Z-Z junction, to determine the transition free energy 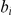 associated with each base pair.

It is energetically most favorable for purines and pyrimidines in the Z-form to be in the *syn* and the *anti* states, respectively. There is a substantial energy cost for a base pair to have the opposite conformation. Although in principle every dimeric sequence can be driven into Z-form, four (CG, GC, CA = TG, and AC = GT) have substantially lower transition energies than do the others. The equilibrium distribution will be dominated either by the untransformed state or by states in which transition occurs at sequences composed of the energetically most favored dinucleotides.

The total free energy of a state 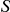 that has 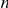 specified base pairs comprising 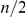 dinucleotide repeat units in 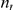 runs of Z-form is given by
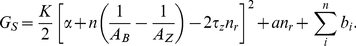
(9)The precise manner in which the base pair transition energies 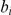 are determined from Table 1 is described below.

### The Strategy of the SIBZ Algorithm

We evaluate the equilibrium B-Z transition in a negatively supercoiled DNA molecule by appropriately modifying the previously developed SIDD method, which has been described elsewhere [45], [49], [51], [55]. Briefly, one first finds the lowest energy state of the system, whose free energy is denoted by 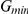. Then one sets an energy threshold 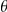, and finds all states whose free energy does not exceed 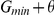. This is done by an exhaustive enumeration procedure [45], [49]. From these states one calculates an approximate partition function and the equilibrium ensemble average values of all quantities of interest.

Since the population of a state at equilibrium decreases exponentially as its free energy increases, the states that are neglected because their energies exceed the threshold are occupied with very low frequencies. If, for example, the threshold is set at 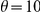 kcal/mol, as is used below, the neglected states are occupied no more than 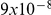 times the frequency of the lowest energy state at 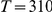 K. A careful density of states calculation was performed to assess the aggregate influence of the neglected high energy states, and to approximately modify the calculated ensemble averages to account for them [49], [51]. Although this correction could not be performed on the transition probabilities of individual base pairs, this approach showed that all other ensemble averages were accurate to between four and five significant figures. Subsequently, we developed an exact, but very slow algorithm that performs these calculations [50]. By comparing its results with those of SIDD at different energy thresholds it was determined that, when thresholds in the range 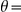
10 to 12 kcal/mol were used, the accuracy of all calculated parameters, including the transition probabilities of individual base pairs, exceeds four significant digits [45].

Since there is a substantial nucleation energy 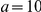 kcal/mol associated with opening each run [21], [36], [40], states with numerous Z-runs will be correspondingly less populated. This allows us to impose a cutoff on the number of runs that may occur. In the SIDD analysis of strand separation it was found that three run states were the most that were encountered at any reasonable energy threshold 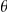 and superhelix density 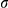. However, sequences that are energetically most susceptible to the B-Z transition tend to be shorter than the A+T-rich regions that favor denaturation. As shown by the energies in Table 1, there are many very costly types of “imperfections” which may hinder the extension of a Z-DNA run. So in the B-Z transition it can be energetically less expensive to initiate a new run at another favorable site than to extend an existing run into an energetically unfavorable region. Extensive sample calculations performed in tuning the SIBZ algorithm have shown that limiting consideration to states with a maximum of four runs is sufficient for high accuracy at the superhelical densities and sequence lengths of interest in this paper.

One important step in the SIBZ algorithm is the assignment of transition energies 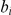 to the dinucleotide repeat units in each Z-run. This is complicated because the transition energy associated to each unit can have any of three significantly different values depending on the *anti* and *syn* characters of the base pairs in that unit and in its neighbors. Briefly, within each unit one base pair must be in *syn* and the other in *anti*. Also there is a significant energy penalty assessed in cases where there are Z-Z junctions with one or both neighbors.

In the SIBZ algorithm we use the following procedure to assign the energetically most favorable *anti* or *syn* conformations to all base pairs in a potentially Z-forming run. First, since the unit cell of Z-DNA is a dinucleotide, we allow only an even number of base pairs in any Z-run. We assign all purines in the run to be *syn* and all pyrimidines to be *anti*, as the dinucleotides with the lowest transition energies have this character. (See Table 1.) Since in most cases forming a Z-Z junction is more costly than flipping a base pair into its non-favorable conformation, we next change the conformation of a base pair if it has the same *anti* or *syn* character as both of its nearest neighbors. This procedure eliminates any repetition of the same conformation (*anti* or *syn*) longer than two base pairs. Then we search for quartets of the form AASS or SSAA. In these we flip the central two bases so that an alternating *syn-anti* character is obtained. Finally, when two bases within a dinucleotide unit have the same conformation, which is not permitted in Z-DNA, we flip the base pair which yields the minimum Z-Z junction energy with its neighbor. There is an ambiguity in this procedure for internal runs of even length at least four, such as ASSSSA, due to the order in which they are flipped. However, the states involved are always high energy because they will have at least one Z-Z junction as well as at least two unfavorable dinucleotides.

To determine the B-Z transition energies of a region, we scan its sequence by dinucleotide units, adding energies of *syn-anti* or *anti-syn* pairs according to Table 1. Whenever the alternating *anti-syn* character is disturbed we add the appropriate Z-Z junction energy. Also, we set the minimum length of a Z-run to be eight base pairs, as shorter regions have not been seen experimentally to form Z-DNA [21]. In this way we assign a B-Z transition energy to each segment in the DNA sequence of even length between 8 and 250 base pairs, which is a reasonable maximum cutoff for a Z-run length.

The algorithmic strategies for finding the lowest energy state, identifying all states that satisfy the threshold condition, and analyzing them to determine equilibrium values of parameters of interest are the same as those developed previously in the SIDD algorithm [45], [49]. We treat circular sequences as described there. A linear sequence is circularized by joining its end to its start with 50 T bases, and the resulting sequence is treated using the same method. In this case we do not report the information for the augmenting 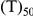 segment. These issues and methods have been described previously [45], [49], [51].

To assess its performance characteristics, we used SIBZ to analyze the pBR322 plasmid (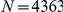 bp) using an energy threshold of 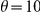 kcal/mol. At superhelical density 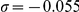 this analysis took 0.12 minutes to run on a MacBook Pro with dual Intel processors. On average there were 23.9 Z-form base pairs and 2.3 runs of transition. A total of 15,829,349 states were found to satisfy the energy cutoff condition 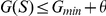. At superhelical density 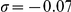 this analysis took 2.25 minutes to run on the same machine. In this case there were averages of 44.2 Z-form base pairs and 3.9 runs of transition, and 1,047,067,293 states satisfied the energy cutoff condition. We find that the execution time is almost constant at superhelix densities 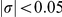, and increases quadratically thereafter. The algorithm scales approximately linearly with sequence length. These performance characteristics suggest that the SIBZ analysis of the complete human genome at 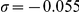 would take approximately 12 hours on a 100 CPU cluster of slightly faster (viz. Opteron) processors, if the sequence was partitioned into 5 kb segments that were analyzed individually. A similar analysis at 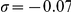 would take approximately ten days.

We note that there is substantial variability of execution speed depending on the attributes of the sequence being analyzed. SIBZ executes quickly on sequences that have one dominant Z-susceptible site. However, the analysis under identical conditions of a sequence in which there is substantial competition among numerous sites can take up to ten times longer.

### Other Methods

*Z-Hunt* was the first algorithm to predict Z-forming regions in DNA sequences based on energy considerations [35]. Although the original version only accepted sequences shorter than 1 Mbp, recently *Z-Hunt* 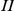 was implemented to identify potential Z-forming regions in longer sequences, and specifically in the human genome [48]. In both versions of *Z-Hunt* a series of fixed length segments within a sequence are separately tested for their Z-forming potential. This is done by inserting the segment in a standard background, which is a circular plasmid in which the inserted segment is the only site that can undergo a structural transition. *Z-Hunt* then calculates the propensity of the segment to form Z-DNA under these standardized conditions. A *Z-score* is assigned to each segment by comparing its ability to adopt Z-form with those of a collection of randomly generated sequences. Unlike in SIBZ where we assign a superhelical density, *Z-Hunt* bases its *Z-score* on the superhelix density at which onset of transition occurs in this standard background. So there is no direct relationship between a segment's *Z-score* and its probability of transition at a specific superhelix density. *Z-Hunt* also provides no information about the competition among multiple Z-susceptible regions within the sequence.

*Z-Catcher* uses a different approach to identify sites with Z-forming potential [47]. This algorithm includes a superhelix density as one of its inputs [47]. It treats the B-Z transition as a simple binary, “on-off” process at a single site. A critical threshold superhelix density is calculated for each individual segment of the sequence being analyzed, at which the energy required by the B-Z transition of that site exactly balances the stress energy released from this transition when it occurs alone in a standard background. If the input superhelix density is more negative than this critical 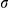, the region is said to be Z-forming. Its output is a list of predicted Z-forming sites, with no weight or probability assigned to them. *Z-Catcher* analyzes individual sites as though complete transition at that site is the only possibility. No consideration is given to how each site competes with all other sites having Z-forming potential within the rest of the sequence. This algorithm is purely mechanical; it does not analyze the equilibrium behavior of the sequence.

SIBZ is the only method developed to date that analyzes the fully competitive B-Z transition behavior of DNA sequences *in situ* at thermodynamic equilibrium under any level of negative superhelicity. It is the only approach that calculates the equilibrium probabilities of transition for each base pair under the given conditions. This provides a more realistic and rigorous analysis, and enables more direct comparisons to be made between its predictions and experimental results than are possible with either *Z-Hunt* or *Z-Catcher*.

## Results

We have applied the SIBZ algorithm to analyze a variety of DNA sequences for their Z-forming properties. These include artificial sequences designed to investigate attributes of the transition, as well as sequences from bacteria and from eukaryotes. We use SIBZ to calculate ensemble average numbers of Z-form base pairs and runs of Z-DNA, as well as the transition profile, which is the graph of the equilibrium probability for transition of each base pair in the sequence. These calculations illustrate how the transition properties vary with superhelical density, and allow comparisons with experimental results and with the results from other methods. Finally, we calculate the propensity for Z-forming regions to be found near the transcription start sites of 12,841 mouse genes.

### The Competitive Nature of Stress-Driven Transitions

The B-Z transition behavior of susceptible regions within a DNA sequence can vary in complicated and highly interdependent ways. This complexity arises because superhelical stresses globally couple together the transition behaviors of all base pairs that experience them. When one region undergoes transition, the change of twist involved fractionally relaxes the level of stress experienced by all other base pairs in the domain. This can be seen from Eq. (7), where a change in the number 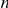 of transformed base pairs causes a corresponding change in the residual superhelicity 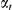 experienced by the entire domain. In consequence, the transition behavior of each base pair is affected by the transformation of any other base pair. This global coupling is the primary reason why superhelical transitions cannot be understood by studying individual sites in isolation, but must be considered in their actual context.

**Figure 1:**
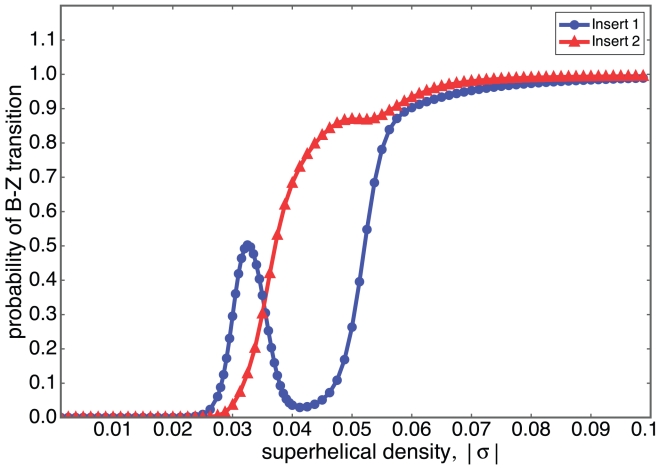
The competition is shown between two segments inserted at separated locations in a 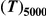 circular plasmid. Insert 1 is 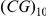, and insert 2 is 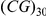 with six Z-Z junctions. The probabilities of each segment flipping entirely to Z-form are shown as a function of negative superhelical density, here plotted as 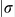.

This competition between different Z-forming regions within a sequence can lead to a rich repertoire of complex, interactive behaviors. We illustrate this with sample calculations on a designed sequence containing two regions susceptible to Z-DNA formation. This sequence consists of 5000 T base pairs, into which we insert two Z-susceptible regions at distant locations. The thymidine background is chosen to insure that only our inserted segments are susceptible to Z-formation. The first insert is a 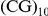 segment while the second is a 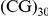 segment that contains six Z-Z junctions. The 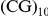 segment is less costly to transform, because the Z-Z junctions in the 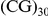 segment are energetically expensive. However because it is shorter, transition at the 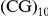 segment also relieves less superhelicity. We used SIBZ to calculate the probability of transition of each of these regions over a range of negative superhelix densities. Plots of these probabilities as a function of 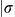 are shown in Fig. 1.

Just beyond the onset of transition where 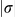 is still small, the superhelical free energy of the untransformed state also is relatively small. Under these circumstance the energy relief afforded by transition is less than it is at more extreme superhelicities. So in this regime the magnitude of the transition energy is the dominant factor in determining which regions transform. This is why the shorter but energetically less costly Insert 1 is the first to transform, as shown in the figure. As 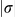 increases the superhelical free energy becomes quadratically larger. Now transitions at longer sequences become more desirable because they relieve more superhelical stress energy. Under these circumstances the difference in transition free energy due to the ZZ-junctions becomes less important than the benefit afforded by transforming a substantially longer segment. For this reason a coupled transition-reversion event occurs around 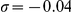, in which transition of Insert 2 is coupled to the reversion of Insert 1 back to B-form. In the range 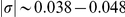 it is energetically too costly for both segments to transform to Z-DNA simultaneously, so such states occur infrequently at equilibrium. Transition of the long Insert 2 has caused substantial relaxation,which decreases the residual superhelicity felt by Insert 1 below the value that would drive it to transform. So at these stress levels the probability of transformation of Insert 1 drops to near zero. As 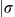 increases beyond the point where Insert 2 has a high probability of being entirely in Z-form, the additional stress accumulates as negative residual superhelicity. When this reaches a sufficient level Insert 1 again transforms to Z-DNA. Beyond 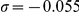 both inserts have high probabilities of simultaneously being in Z-form. One sees that there are specific intervals of superhelicity within which 1) neither insert transforms, 2) the first transforms but not the second, 3) the second transforms but not the first, or 4) both inserts transform simultaneously. The transition in this example experiences every logical possibility.

When *Z-Hunt* is applied to this sequence it assigns *Z-score* of 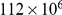 to Insert 1, and 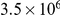 to Insert 2. The above analysis shows that when the competition between sites is considered there are circumstances when a region with a lower *Z-score* may transform while one with a higher *Z-score* does not. The analysis of individual sites in isolation simply does not capture the complexity of behavior that can occur in stress-driven transitions.

### B-Z Transitions in Sample DNA Sequences

**Figure 2:**
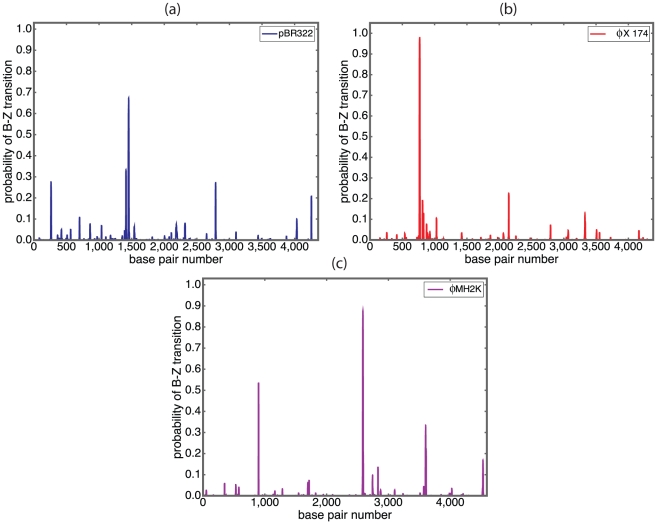
The B-Z transition profiles calculated at superhelical density 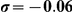 are plotted for three circular genomes: (a) the pBR322 plasmid, (b) bacteriophage 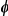
X 174, and (c) Bdellovibrio phage 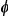
MH2K.

We have analyzed the B-Z transition properties of three circular DNAs - the pBR322 plasmid, and the 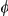
X174 bacteriophage and Bdellovibrio phage 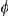
MH2K genomes. Fig. 2 shows the B-Z transition probability profiles of these sequences calculated at superhelical density 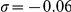. In each case the B-Z transition is substantially confined to a small number of sites where it is energetically most favorable, although there are several additional locations that have smaller, but still significant, transition probabilities. All sites with transforming potential are seen to be relatively short. The longest Z-forming regions found in any of these three sequences at 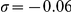 contains 14 base pairs. In each of these sequences there is at least one predominant region whose probability of forming Z-DNA exceeds 70%. In pBR322 the largest peak has probability near 70%, and there are three other sites whose probabilities exceed 25%. Phage 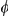
X174 contains a single region, slightly longer than that in pBR322, whose transition probability is close to unity. Because this region is so dominant at this superhelix density, other portions of this sequence have only low probabilities of Z-formation. This dominance is a consequence of this site having a highly favorable transition energy over a sufficiently long region.

We compared the performance of SIBZ with those of *Z-Hunt* and *Z-Catcher* when run on these sequences [35], [47], [48]. The energies from Table 1 were used in all three programs. When *Z-Hunt* was applied to the pBR322 sequence it found one 15 bp long segment at location 1448 with a *Z-score* of 2444, and a 17 bp long segment at position 1407 with a *Z-score* of 1845. As shown in Fig. 2a, SIBZ also finds these two peaks to be the most dominant, with the segment at location 1448 having the higher transition probability. This agrees with the relative *Z-score* rankings provided by *Z-Hunt*. However, the relative probabilities of these two regions are not proportional to their *Z-score*. Moreover, SIBZ documents several other regions that also have significant transition probabilities that *Z-Hunt* does not identify. One sees that, although the sites found by *Z-Hunt* agree with the major sites found by SIBZ, the latter provides more information regarding other Z-susceptible regions. The results from SIBZ, because they are expressed as transition probabilities of the fully competitive transition at the assumed superhelix density, are both more precise and more easily interpretable than are *Z-scores*.

*Z-Catcher* does not identify any Z-susceptible regions in pBR322 at superhelix density 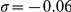. At superhelix density 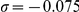 it finds the two dominant segments at locations 1407 and 1448. It also finds two other Z-segments at positions where no Z-forming potential is seen by the other algorithms. The segment at position 1448 is found to comprise 28 base pairs, which is significantly longer than predicted either by *Z-Hunt* or by SIBZ. Thus, the predictions of *Z-Catcher* seem to differ considerably from those of either *Z-Hunt* or SIBZ.

**Figure 3:**
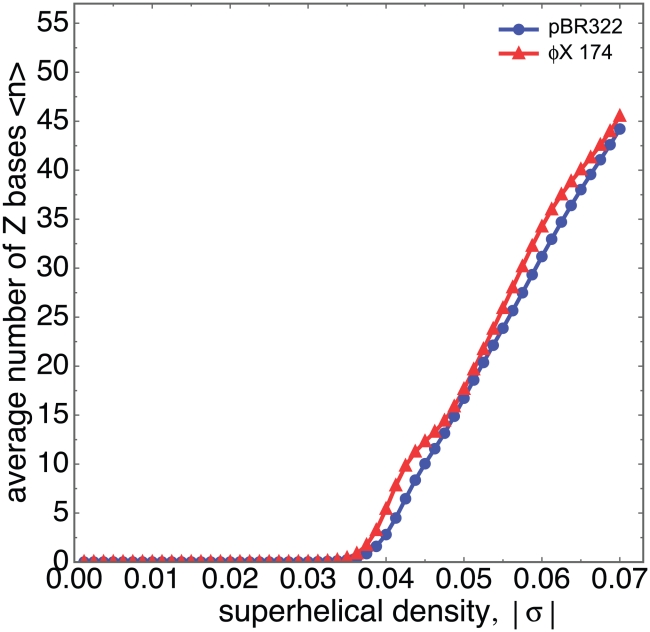
The average number 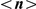 of Z-forming base pairs is plotted as a function of superhelical density 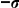 for pBR322 and 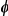
X-174.

The behavior of a B-Z transition varies significantly as the negative superhelical density is modified. In general, as 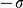 is increased, larger numbers of transformed base pairs are required to relieve torsional stresses. If the most energetically susceptible region is sufficiently long, the most favored way to do this would be by extending the transition to encompass increasing amounts of this region. However, the most Z-susceptible regions in natural DNA sequences are usually relatively short, as is seen in these three sequences. In that case what commonly happens is that, as 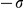 (and hence also the level of imposed stress) increases, successively more energetically costly distinct Z-forming regions are transformed (possibly coupled to reversion of other regions as shown above). In either of these strategies the average number of base pairs adopting the Z-form increases with negative superhelical density. This is demonstrated in Fig. 3, which shows the average number 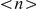 of Z-DNA base pairs as a function of 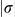 for pBR322 and for 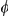
X174. One sees that in both sequences there is a threshold for the onset of transition around 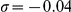, and the expected number of transformed base pairs increases approximately linearly thereafter. (We note that the onset of transition in Fig. 1 occurs at a slightly less extreme superhelix density than is seen in Fig. 3. This occurs because according to Table 1 the shorter Z-susceptible insert in the constructed sequence has the lowest possible transition energy. This is not true of the most Z-susceptible site in either 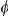
X174 or pBR322.)

### Comparisons with Experiments - Onset of Transition

Z-DNA forming regions have been observed with two-dimensional gel electrophoresis in plasmids engineered to contain Z-susceptible inserts [37], [56]. The susceptibility of a region to be driven into Z-form was found to depend on its base sequence and on the level of supercoiling of the plasmid. In one experiment Peck *et al.* inserted a 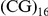 sequence (here called Sequence 1) into the *Bam*HI site of pBR322 [56]. This is the most energetically susceptible sequence to Z-DNA formation, as shown in Table 1. In another set of experiments pBR322 derivatives were analyzed, each of which contains an insert whose sequence includes “imperfections” relative to the optimal 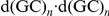 Z-forming segments [37]. Here we focus on two of these engineered plasmids, one with 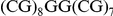 (Sequence 2) inserted into the *Bam*HI site of pBR322 and the other with 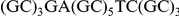 (Sequence 3) inserted into *Pvu* II site of pBR322.

The Z-forming insert sequences 1 and 2 both have the same length, each containing 16 dinucleotides (i.e. 32 base pairs). This means they have the same ability to relieve superhelical strain. However, Sequence 2 contains a Z-Z junction consisting of GG bases, which breaks the alternating *syn-anti* pattern and costs an extra 4 kcal/mol to form (see Table 1). So the onset of transition in Sequence 2 is expected to occur at a more extreme superhelix density because it has a higher total B-Z transition energy. Transition at Sequence 3 has two disadvantages relative to the others. It is shorter in length, containing 13 Z-forming dinucleotides instead of 16, and it has two energetically costly “imperfections”. The GA and TC nucleotides in the *anti-syn* conformation cost 3.4 kcal/mol each, resulting in an additional transition cost of 6.8 kcal/mol relative to a perfect alternating CG sequence. Therefore, the critical superhelix density 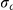 for complete Z-formation of this region is expected to be substantially higher than for either Sequence 1 or 2.

**Figure 4:**
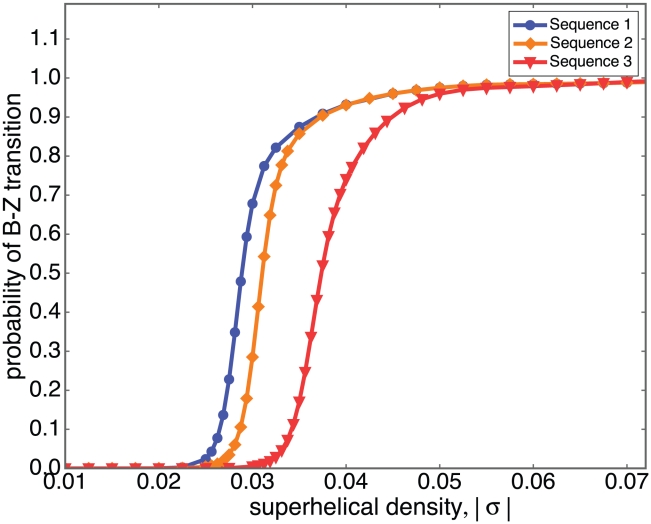
The probability of B-Z transition is plotted as a function of superhelical density 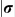 for three pBR322 derivatives with inserted Z-susceptible sequences. Sequence 1 has sequence 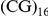 inserted into the *Bam*HI site of pBR322. Sequence 2 has 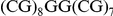 inserted at the same location. Sequence 3 has 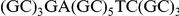 inserted into the *Pvu* II site of pBR322. In the text these results are compared with those from experiments performed by Peck *et al.* [56] and by Ellison *et al.* [37].

Fig. 4 shows the probabilities predicted by SIBZ of the inserted sequences transforming to Z-DNA in each of these three pBR322 derivatives, plotted as a function of superhelical density 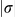. The curves labeled Sequence 1, Sequence 2 and Sequence 3 refer to the plasmids with those as their inserts.

Experimentally measurements have been made of the critical superhelical density 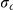 required to flip the entire inserted sequence into Z-DNA [37], [56]. These analyses regarded the transition as two-state, analogous to an “on-off” switch, and determined the critical superhelicity at which these inserted segments were switched on. These critical densities 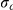 are shown in column 3 of Table 2.

**Table 2:** Onset of transition: Comparison of experimental results with SIBZ.

Sequence 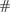	Inserted Segment	Experimental 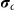	Calculated 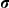 at 80% Probability
1	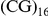	−0.031 [56]	−0.032
2	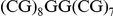	−0.034 [37]	−0.034
3	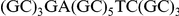	−0.042 [37]	−0.041

Critical superhelical densities 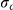 for the onset of a B-Z transition are obtained experimentally for three sequences in which Z-forming regions are inserted [37], [56]. These results are compared with SIBZ, where we define a region to be in Z-form when its probability of transition is 80%.

It is difficult to make exact comparisons between our theoretical predictions and these experimental results because they do not involve entirely comparable quantities. The experiments measure a critical 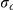 for completion of an “on-off”, two-state B-Z transition, while SIBZ calculates the equilibrium probability of the plasmid experiencing any amount of transition. However, the trend observed in the SIBZ results of Fig. 4 closely agrees with experimental data. Transition occurs at the least extreme superhelix density in the plasmid with the Sequence 1 insert, later in the plasmid with Sequence 2 insert, and lastly in the plasmid with the Sequence 3 insert. Moreover, the horizontal displacement between these curves is about 0.0025 between Sequence 1 and Sequence 2, and about 0.008 between Sequence 2 and Sequence 3. These agree closely with the experimentally measured differences.

In order to directly compare the experimental finding with the SIBZ results we must decide what level of transition corresponds to the “on-off” switch having been thrown. We define this to occur when the probability of Z-form is 80%. Column 4 in Table 2 shows the superhelix densities at which this occurs for each of the three inserts, as determined from the curves in Fig. 4. At this level SIBZ results are seen to agree closely with the experimentally measured 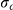 values.

### Comparisons with Experiments - Sites of Transition

We next compared the predictions of SIBZ with the results of antibody binding experiments that identified Z-forming regions in the human c-*myc* gene [29], [30]. Here the formation of Z-DNA is driven by the negative superhelicity that is generated when the gene is transcribed. This was confirmed by the observation that Z-formation was suppressed when transcription was inhibited.

**Figure 5:**
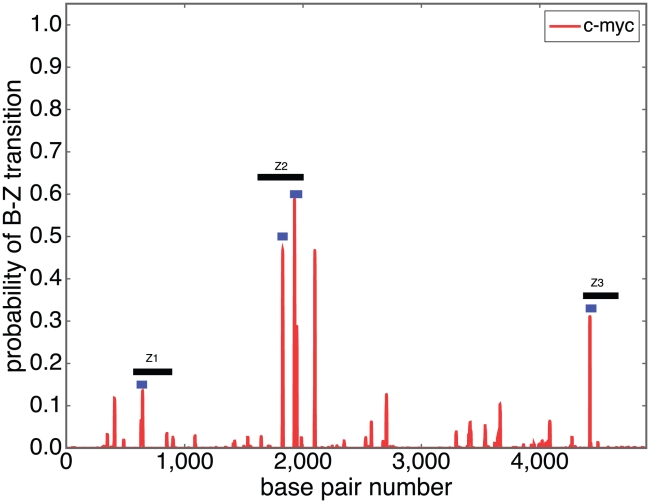
The SIBZ profile is shown for the 5 kb region of the human c-*myc* gene that was shown experimentally to contain Z-forming regions [29], [30]. The three experimentally characterized Z-forming segments are labeled Z1, Z2, and Z3. The specific Z-forming regions within each of these segments are shown by short, blue bars [30].

The first experiment used anti-Z antibodies to isolate regions of Z-DNA formation [29]. They found three restriction fragments from the c-*myc* gene region that showed antibody reactivity when the gene was being transcribed. These three fragments are all located in the upstream region of the gene in proximity to its three promoters. Fig. 5 shows the SIBZ transition profile of a 5kb region around the c-*myc* gene that spans these locations. This profile was calculated at superhelical density 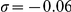, a reasonable value for transcriptionally driven superhelicity [6], [7]. The three large, gray horizontal bars labeled Z1, Z2, and Z3 identify the segments that were found in this experiment to contain Z-forming regions [29]. Five of the six sites identified by SIBZ as having the highest Z-forming probabilities occur within these three segments. The peak that is not within any of the three experimentally identified fragments is located around position 2100 in Fig. 5. Perhaps this region was missed because of its proximity to the Z2 fragment.

A second experiment identified the exact locations of the Z-forming sites within these three segments Z1, Z2, and Z3 [30]. This was done by isolating the fragments and inserting each individually into the circular pDPL6 plasmid [22]. Regions of Z-DNA were detected by measuring diethyl pyrocarbonate reactivity within these negatively superhelical constructs. One Z-form region was found experimentally in each of the Z1 and Z3 segments, and two such regions were found in Z2. The experimentally determined locations of these regions are shown in Fig. 5 by small, blue horizontal bars located below each larger, labeled segment. These regions coincide precisely with the strongest Z-forming sites predicted by SIBZ. In each segment all the sites that are predicted to be most Z-susceptible are found experimentally to actually be in Z-form. SIBZ correctly identifies all sites within the three segments that occur in Z-form, with no false positives. This shows that our theoretical model produces results that agree precisely with those obtained by experiments.

### Z-Susceptibility around Transcription Start Sites in Eukaryotes

**Figure 6:**
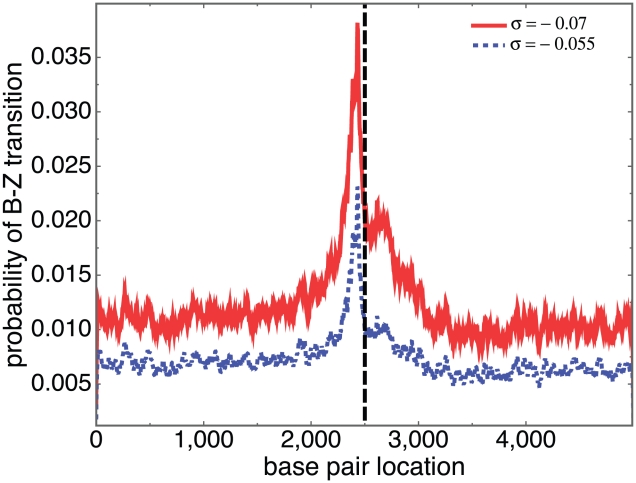
The average probability of B-Z transition is plotted as a function of base pair location, the average being taken over 12,841 mouse genes. The sequences were aligned so their TSSs are all located at position 2500, indicated by the vertical dashed line. The solid and dotted lines are the results for 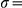
−0.07 and −0.055, respectively.

The experiments described above on the c-*myc* gene suggest that Z-forming regions may occur in proximity to transcription start sites. The accuracy of SIBZ in identifying these regions allows us to use it to address this question. To this end we analyzed 5 kb regions around the transcription start sites (TSSs) of 12,841 mouse genes. We oriented all genes to read left to right, and aligned them so their TSSs were at position 2500. We calculated the probability of B-Z transition at each of the 5,000 positions in each sequence. We then averaged the transition probabilities at each position that were calculated for all the sequences. This analysis was performed at two superhelix densities, 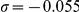 and 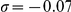. The results are shown in Fig. 6.

A substantial enrichment of predicted Z-form sites is observed immediately upstream of the TSS at both superhelix densities, with the number of these sites increasing with 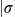. The number of predicted sites immediately downstream from the TSS is approximately half the maximum number immediately upstream. The density of predicted Z-form regions in the far upstream, inferentially intergenic portions of the sequences approximately equals that in the far downstream, inferentially transcribed regions, and is approximately 30% of the maximum at both superhelix densities.

To analyze this data further we define a region within an individual sequence as Z-forming if its probability of B-Z transition exceeds 80%. At superhelix density 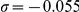 a total of 2519 of the 12841 genes (19.6%) are found to have one or more Z-forming regions within 1,000 bp upstream of (i.e. 5′ to) their TSS. At the more extreme superhelix density of 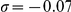 this number increases to 4269 (33.2%). There is a clear enrichment of Z-forming regions directly upstream of TSSs relative to downstream. At 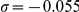 there are 1083 genes that have one or more predicted Z-forming regions within 50 base pairs upstream of the TSS, and 572 genes with such a site within 50 base pairs downstream, a nearly two-fold change. At 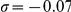 the corresponding numbers are 2077 genes and 1304 genes, respectively. We also find that the majority of these 12,841 mouse genes contain Z-susceptible regions somewhere within their 5 kb regions. At 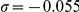 we observe that 32.7% of these genes do not contain a Z-forming region (at the 80% level) anywhere in their sequence. This percentage drops to 12.7% when 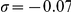.

A similar analysis was performed by Droege using *Z-Catcher* to analyze a large collection of human genes [47]. That work documented a similar enrichment of predicted Z-susceptible regions in the 5′ flanks of genes. Our results confirm and reinforce theirs, although the methods used in the two analyzes are not equivalent.

## Discussion

In this paper we have developed the first statistical mechanical method to analyze the competitive B-Z transition within long superhelical DNA domains of specified base sequence. The output of this method is the probability of transition calculated for each base pair in the sequence, rather than simply a list of sites or a *Z-score*. We have demonstrated the essentially competitive character of these transitions, and shown how the transition behavior can change in complicated ways with the level of imposed superhelicity. We find that there are regions with clear Z-forming potential in genomic DNA sequences. Our results agree in detail with experimental measurements of both the onset of transitions and the locations of Z-forming regions.

Our analysis of 12,841 mouse genes documents a substantial increase in the occurrence of potential Z-forming regions immediately upstream from transcription start sites. At a superhelical density of 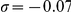 we find that 33.2% of these genes have one or more Z-forming regions within 1,000 bp upstream of their TSS. Approximately half of these have such a site within 50 bp 5′ of the TSS. We note that in eukaryotes this superhelical density is attained in these regions through transcription-driven superhelicity [7]. This suggests that Z-DNA could play roles in transcriptional regulation. However, the fact that less than half the genes have Z-forming regions in their immediate 5′ flanks (i.e. within 1 kb of the TSS) suggests that there may be distinct classes of genes, some of which use such sites for regulatory purposes while other do not. Also, since Z-forming regions are relatively short, around 14 base pairs, there is room for them to be interspersed with other motifs, including A+T rich regions. These issues will be addressed more fully in a subsequent paper.

When we compare the results of SIBZ with those of *Z-Hunt* and *Z-Catcher* we find that *Z-Hunt* and SIBZ agree in identifying the most dominant sites. However, SIBZ also identifies several sites where the probability of transition remains significant, that neither of the other methods find. *Z-Catcher* seems to be less sensitive than either of the others, only identifying the most susceptible sites when its input superhelix density is relatively large. Neither *Z-Hunt* nor *Z-Catcher* analyze the actual B-Z transition behavior of a superhelical DNA sequence, which is competitive and can vary in highly complex ways with the superhelix density. Instead they seek only to identify those individual sites within the sequence that have the greatest potential to form Z-DNA. They say nothing about how these sites compete, and provide no information about transition probabilities that can be directly compared with experimental results.

We note, however, that *Z-Hunt* and *Z-Catcher* still may prove useful for specific purposes. In particular, in our experience each is substantially faster than SIBZ. It is difficult to compare their relative speeds, especially when they are only accessible through websites. However, both *Z-Hunt* and *Z-Catcher* appear to return results on 5 kb sequences almost instantaneously, whereas SIBZ takes from ten seconds to three minutes depending on conditions and sequence characteristics. Since it seems to identify the major sites reasonably well, *Z-Hunt* in particular may serve as an initial screen for such sites, with SIBZ used to perform full analyses as needed.

The methods presented here can be applied to any two-state transition, provided the geometry, deformability, and transition energetics of the states are known. In this regard the best characterized DNA transition is strand separation. Both enthalpies and entropies of denaturation have been measured for every base pair and every choice of its nearest neighbors. The dependence of the free energy on ionic strength also is known. Together these allow one to predict how this transition behavior will vary with changing ionic conditions and temperature, as well as with superhelicity.

At present the energetics of the B-Z transition are not so well characterized. It is known that specific alternating purine-pyrimidine sequences are substantially favored for Z-formation, and their transition free energies have been measured. But no quantitative information is currently available regarding how these free energies partition between entropy and enthalpy, nor about their ionic strength dependences. However, it has been reported that the B-Z transition shows little temperature dependence in the range between 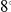
C and 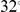
C [57]. This suggests that entropy changes are much smaller for B-Z transitions than for denaturation, where the DNA is disordered and its interactions with the solvent are thereby substantially altered. The extremely close accord documented here between the predictions of SIBZ and experimental results suggests that the transition energetics we use are accurate. We note that this accuracy is achieved without having any tunable parameters in our model.

Although SIBZ is effective at analyzing the B-Z transition behavior of a supercoiled DNA molecule, it still focuses on only part of the complete picture. It is well known that local strand separation at A-T rich regions also occurs in negatively supercoiled molecules. To enable the accurate analysis of the full transition behavior of superhelical DNA one must include both types of transitions in a unified model. This would permit all sites susceptible to SIDD and/or to B-Z transitions to compete. We are working to develop such a model.

A website is available (http://benham.genomecenter.ucdavis.edu) where the members of the scientific community may submit sequences of interest to them for analysis by the SIBZ algorithm. The sequence must be either in FASTA format or in a file that contains sequence characters exclusively. Sequences of any length up to 10 kb may be submitted, although sequences of length around 5 kb are preferred. This site may also be used for SIDD analysis of the same sequences.

In the near future we hope to analyze the SIBZ characteristics of a large number of genomic sequences, up to and including complete genomes of model organisms. The results for each sequence will be posted in a database on the same Web site as they are completed.
